# 3D versus 4K laparoscopic vaginal cuff closure after hysterectomy by surgeons in training: a prospective randomised trial

**DOI:** 10.52054/FVVO.16.3.029

**Published:** 2024-09-30

**Authors:** M Pavone, S Di Berardino, G Esposito, A Baroni, M D’Indinosante, MT Giudice, A Gioé, F Campolo, U Catena, G Scambia, F Fanfani, S Restaino

**Affiliations:** Dipartimento di Scienze per la Salute della Donna e del Bambino e di Sanità Pubblica, Fondazione Policlinico Universitario A. Gemelli, IRCCS, UOC Ginecologia Oncologica, Rome, Italy; IHU Strasbourg, Institute of Image-Guided Surgery, Strasbourg, France; Università Cattolica del Sacro Cuore, Rome, Italy; Department of Medical Area (DAME), University of Udine, Clinic of Obstetrics and Gynecology, “Santa Maria Della Misericordia” University Hospital, Azienda Sanitaria Universitaria Friuli Centrale, Udine, Italy

## Abstract

**Background:**

Technological advances in visual systems have contributed to overcoming the limitations in spatial perception of minimally invasive techniques. To date, there is a lack of literature on the advantages of 3D vision systems over 4K in laparoscopic surgery, although benefits have been observed in the training setting.

**Objectives:**

To compare operating times, perioperative outcomes, and task achievement using 3D and 4K vision systems for vaginal cuff closure performed by residents during total laparoscopic hysterectomy (TLH). All surgeons in training have obtained the Gynaecological Endoscopic Surgical Education and Assessment (GESEA) certificate.

**Materials and Methods:**

This is a prospective randomised trial (NCT04637022). Women undergoing total hysterectomies for benign conditions between January 2021 and November 2023 were enrolled in the study. Vaginal cuff closures were performed by surgeons in training who had obtained the second level of the GESEA programme certificate.

**Results:**

Fifty-four patients were enrolled. There were no statistically significant differences in time between 3D and 4K vision for vaginal cuff closure (p=0.918). No statistically significant differences were observed for mean estimated blood loss (EBL) (overall: 62.85 ± 22.73mL; 3D: 65 ± 24.83mL; 4K: 61.11 ± 21.18; p=0.556) and median hospital stay (p=0.234). Three non-severe intraoperative complications in the 3D group (p=0.048) and three postoperative complications in the entire cohort (p=0.685) were reported.

**Conclusion:**

The operating time for vaginal cuff closure performed by trainee surgeons is similar when comparing 3D vision during conventional laparoscopy and 4K vision systems. The choice of surgical vision systems may be guided by a cost analysis and surgeon preferences.

**What is new?:**

Substantial evidence is lacking regarding the advantages of incorporating 3D vision into standard laparoscopy for gynaecological surgery. This research seeks to assess whether the 3D visual system can provide benefits as compared to 4K visualisation during laparoscopic vaginal cuff closure performed by surgeons in training within the GESEA 2 certification programme.

## Introduction

The first total laparoscopic hysterectomy (TLH) was pioneered by Harry Reich in 1989 (Reich, 1992). Since then, the advent of minimally invasive surgery (MIS) has significantly reduced the prevalence of open abdominal hysterectomies (Gueli Alletti et al., 2020). This shift has been driven by consistently related benefits of MIS over laparotomy (Guo et al., 2023).

The first series reporting laparoscopic vaginal closure after TLH addressed the potentially increased incidence of vaginal cuff dehiscence as compared to cuff suturing with the vaginal approach (Kim et al., 2014; Uccella et al., 2011). Technological and procedural advances over the years have improved surgeon proficiency, as laparoscopic suturing with intracorporeal knots is now considered the preferred approach for vaginal cuff closure at the end of laparoscopic hysterectomy (Uccella et al., 2018).

The evolution of vision systems over the last two decades has significantly contributed to make up for the lack of depth perception in MIS as compared to open surgery. The advent of tridimensional (3D) vision camera attempted to address this shortcoming using dual channel stereo endoscopes and passive polarising stereoscopic projection which can generate high-quality 3D images (Wenzl et al., 1993). However, when using 3D vision, eye fatigue, blurred vision, difficulty in focusing, and nausea have been reported as side effects (Schwab et al., 2017). In the meantime, the introduction of high- definition/ultra-high-definition (4K) scopes into the marketplace has allowed magnified views with substantial details without the distress associated with 3D scopes. Despite such advancements, suturing the vaginal vault remains one of the most challenging laparoscopic tasks, particularly in the early stages of training (Ajao et al., 2020). Intracorporeal needle loading, driving, and reloading necessitate extensive rotational wrist movements, dexterity, spatial vision, and depth perception (Mereu et al., 2013). The rise of robotic surgery is attempting to overcome such challenges (Restaino et al., 2020). Robotic arms are equipped with articulating instruments which do not only surpass the range of motion of the rigid laparoscopic ones, but which can also exceed that of human wrists through clutching (>360-degree rotations). Additionally, platforms offer 3D vision either with deep immersion closed-console systems or in an open environment with flat screens (Pavone et al., 2023).

The European Association for Endoscopic Surgery (EAES) recently issued recommendations endorsing the use of 3D systems in clinical settings to reduce operating times (Arezzo et al., 2019). However, there is a notable gap in clinical trials examining whether 3D systems offer performance benefits over ultra-high-definition 4K laparoscopic imaging. The primary endpoint of this prospective study was to verify whether the operating time required to perform vaginal vault closure during TLH by surgeons in training can be reduced using 3D laparoscopy as compared to 4K. The secondary endpoints were to evaluate differences concerning the incidence of intraoperative and postoperative complications and the completion rate of the task by surgeons in training who achieved the GESEA 2 certification programme.

## Patients and methods

### Patients and study participants

This is a prospective randomised clinical trial conducted at Fondazione Policlinico Universitario Agostino Gemelli, IRCCS, in Rome (Italy). Prior to starting patient enrolment, the study protocol obtained the approval from the internal Ethics Committee (protocol number 0043736/20 ID:3558) and was registered on the clinicaltrial.gov platform (NCT04637022).

From January 2021 to November 2023, patients undergoing laparoscopic hysterectomy were actively recruited for the study. Inclusion criteria encompassed the following: i) a preoperative diagnosis of a benign uterine condition (such as uterine fibroids, abnormal blood loss, complex hyperplasia with atypia, uterine prolapse) warranting total hysterectomy, ii) uterine size ≤ 15cm upon preoperative assessment, iii) body mass index (BMI) < 30, iv) American Society of Anaesthesiologists (ASA) class ≤ 2, and v) absence of pregnancies or pelvic inflammatory disease at the time of the study. Patients with a preoperatively suspected neoplastic pathology or those ineligible for surgery were excluded. Informed consent was signed preoperatively by all women allowing their data to be collected and analysed for scientific purposes. After a lapse of 20 minutes without completion of the surgical task by the trainee surgeon, the overseeing senior surgeon took charge of the procedure and finalised it.

### Surgical procedure

Following a 1:1 randomisation, all patients underwent total laparoscopic hysterectomy (TLH), using either 3D (10mm, 0-degree 3D high- definition laparoscopy; Olympus Winter & IBE GMBH, Hamburg – Germany) or 4K (10mm, 0-degree ULTRA Telescopes; Olympus Winter & IBE GMBH, Hamburg – Germany) laparoscopes. The allocated vision system was used for the entire procedure and not just for vaginal cuff closure.

Patients were not blinded to the vision system used, as it was deemed irrelevant to the study’s investigations.

**Figure 1:**
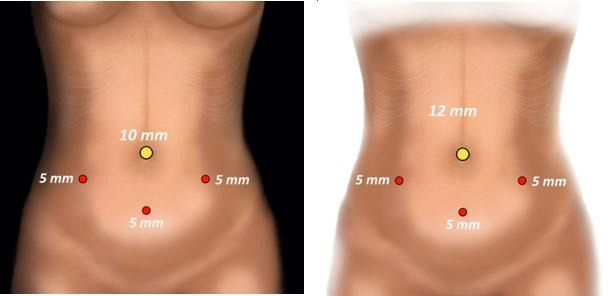
Port placement: Four-port configuration, comprising a 10mm or 12mm optical port at the umbilicus, three accessory ports (5mm each) in the bilateral lower quadrants, and a 5mm port in the suprapubic midline.

Laparoscopic hysterectomy adhered to surgical standards (Gueli Alletti et al., 2020) with a four-port approach, comprising a 10mm or 12mm optical port at the umbilicus, three accessory ports (5mm each) in the bilateral lower quadrants, and a 5mm port in the suprapubic midline (Figure 1). The choice of the port size may be decided upon according to the surgeon’s preferences or depending on the need for specific intra-abdominal device insertion. Once colpotomy was performed and the uterus was extracted, the vaginal cuff closure technique used a braided and coated 0-polyglactin suture on a half-circle HR26 needle, was executed in a continuous fashion. The needle’s path involved the initial passage through the anterior and posterior layer of the left apex of the vaginal cuff, ensuring inclusion of the uterosacral ligament in the initial bite. Subsequently, the thread was knotted, tied, and cut, leaving 2 to 3cm for the final closure at the end. The needle then traversed the right apex, and the thread was knotted and tied. A running suture was continued along the length of the vaginal cuff, maintaining approximately 5 to 10mm between each suture bite.

### Endpoints

The primary endpoint of the study was the total time required to complete the closure of the vaginal cuff, measured in minutes. This duration began from the initial grasp of the suture to its cutting. To prevent any undue prolongation of operating time potentially harmful to patients, a 20-minute allocation was set for the completion of cuff closure by surgeons in training. Beyond this timeframe, the responsibility for this task was transferred to the attending surgeon, with the circulating nurse overseeing and recording the duration of the cuff closure.

Secondary outcomes included perioperative measures, as well as the rate of task completion by surgeons in training. Anonymous data collection encompassed age at surgery, body mass index (BMI), history of previous abdominal and uterine surgeries, parity, estimated blood loss (EBL), days of hospital stay (HS), and the occurrence of intraoperative and postoperative complications.

The Common Terminology Criteria for Adverse Events v3.0 (CTCAE) were used to categorise intraoperative complications (CTCAE 0-1 vs. CTCAE ≥ 2). Early postoperative complications were graded according to the extended Clavien- Dindo classification of surgical complications.

### Surgeons in training

All laparoscopic vaginal cuff closures were performed by residents in their final year of residency, under the supervision of an experienced surgeon. The surgical competency of participating residents was assessed and evaluated by the Principal Investigator (PI) or the Co-PI. The assessment considered their bimanual coordination, tissue handling capacity, and appropriate decision-making based on intraoperative findings. Additionally, all involved residents completed the programme and obtained the Gynaecological Endoscopic Surgical Education and Assessment (GESEA) second level certification and diploma of Minimally Invasive Gynaecological Surgeon (Campo et al., 2016). The GESEA programme is a comprehensive training initiative in gynaecological endoscopy designed to foster both theoretical understanding and hands-on proficiency using pelvic simulators. It serves as the official certification programme endorsed by the European Society for Gynaecological Endoscopy (ESGE). The programme revolves around theoretical knowledge, practical skills, and the assessment of the reached level of competency. The first step involves obtaining the GESEA Bachelor Certificate, affirming a grasp of general endoscopic knowledge and the acquisition of fundamental endoscopic psychomotor skills. The subsequent stage entails the successful completion of the GESEA MIGS (Minimally Invasive Gynaecological Surgeon) exam, leading to the conferment of the GESEA MIGS Certificate. This certification attests that the trainee has not only mastered advanced knowledge and psychomotor skills but also possesses the capability to autonomously perform standard procedures in gynaecology.

### Statistical analysis

The null hypothesis in this study posited that the adoption of 3D cameras for laparoscopic vaginal cuff closure by trainees would result in a shorter operating time as compared to the same procedure performed with 4K laparoscopes. Based on literature-derived information suggesting a mean operating time of 15 minutes for vaginal vault suture (Ajao et al., 2020; Restaino et al., 2021), we calculated that a minimum sample size of 22 participants per arm would be required to achieve a 25% reduction in this time (11.5 minutes), with an alpha error of 0.05 and beta error of 0.2. This sample size was adjusted to 25 participants per arm to account for a potential dropout rate of 10%. To ensure unbiased results, a randomisation computer list was thoroughly reviewed.

Descriptive statistics were used to characterise patients and surgical features. Data normality was verified using the Kolmogorov-Smirnov test. Quantitative variables were described using mean and standard deviation (SD) or median and interquartile range (IQR) for non-normally distributed variables. Qualitative variables were summarised using absolute and percentage frequency tables.

Groups were compared using the ANOVA test or the Mann-Whitney U test for continuous variables and the χ2 test for categorical variables, as deemed appropriate. A p value < 0.05 was considered statistically significant for the two- tailed test. All statistical analyses were performed using SPSS version 29.0.1.0 (IBM, Armonk, New York, United States).

## Results

Fifty-four patients were enrolled and randomised, for vaginal cuff closure using two vision systems (3D/4K). Out of the 54 cases, 26 patients were assigned to the 3D vision group, and 28 to the 4K group. Seventeen residents, who had obtained GESEA2 certification and underwent competency assessments by the Principal Investigator (PI), performed the surgical procedures. In 9% of total cases (4/26 for the 3D group and 1/28 for the 4K group), residents were unable to complete cuff closure within the predesignated 20 minutes, and these patients were subsequently excluded from the statistical analysis.

Demographics and baseline characteristics were comparable between groups, with a median age of 51.5 (IQR: 47-55) years and a median BMI of 23.8 (IQR: 21.6-25.6) for the overall population (Table I). Uterine fibroids were the most common indication for surgery (65.3% in 3D; 60.7% in 4K), followed by hysterectomies for prophylactic purposes (23% in 3D; 17.8% in 4K), atypical endometrial hyperplasia (3.8% in 3D; 7.14% in 4K), and adenomyosis (7.7% in 3D; 3.5% in 4K).

**Table I:** Baseline patient characteristics. BMI: Body mass index. Descriptive statistics are expressed as mean and standard deviation (SD) or median (interquartile range: IQR) for quantitative variables, as absolute and relative percentage frequencies for qualitative variables.

Variables	ALL (n= 54)	3D (n= 26)	4K (n= 28)	p value
Age, years	51.5 (47-55)	50.5 (46-54)	52 (48.5-56)	0.468
BMI, kg/m^2^	23.8 (21.6-25.6)	22.9 (21.7-26.6)	24 (20.85-24.85)	0.993
Previous abdominal surgery				
Laparoscopy (%)	8 (14.8%)	3 (11.5%)	5 (17.8%)	0.513
Laparotomy (%)	28 (51.8%)	14 (53.8%)	14 (50%)	0.777
Parity > 1	40 (74%)	20 (76.9%)	18 (64.28%)	0.376
Indication for surgery				
Fibroids	34 (62%)	17 (65.3%)	17 (60.7%)	
Atypical endometrial hyperplasia	3 (5.5%)	1 (3.8%)	2 (7.14%)	
Adenomyosis	3 (5.5%)	2 (7.7%)	1 (3.5%)	
Prophylactic	11 (20.3%)	6 (23%)	5 (17.8%)	
Other	3 (5.5%)	0	3 (10.7%)	

Mean vaginal cuff closure time was 13.02 ± 3.37 minutes for the overall cohort, with no statistically significant differences between 3D and 4K vision (12.97 ± 3.6 min versus 13.07 ± 3.25 min, p=0.918). Total operating time for hysterectomies was reported as 160.04 ± 47.32 minutes, with no significant differences between the two groups (p=0.498). Similarly, no statistically significant differences were observed for mean estimated blood loss (EBL) (overall: 62.85 ± 22.73mL; 3D: 65 ± 24.83mL; 4K: 61.11 ± 21.18; p=0.556) and median hospital stay (p=0.234).

Regarding intraoperative complications, three non-severe hysterectomy-related complications were reported in the 3D group (p=0.048) with a CTCAE grade < 2. Additionally, three postoperative complications were noted (p=0.685), one in the 3D group (4.5%) and two in the 4K group (7.4%), of these, one was a cuff dehiscence (Table II).

**Table II:** Perioperative details. *Common Terminology Criteria for Adverse Events (CTCAE) Descriptive statistics are expressed as mean and standard deviation (SD) or median (interquartile range: IQR) for quantitative variables, as absolute and relative percentage frequencies for qualitative variables.

Variable	ALL (n= 49)	3D (n= 22)	4K (n= 27)	p value
Total operating time, minutes	160.04 ± 47.32	165.22 ± 52.12	155.81 ± 42.55	0.498
Vaginal cuff closure time, minutes	13.02 ± 3.37	12.97 ± 3.6	13.07 ± 3.25	0.918
Estimated blood loss, mL	62.85 ± 22.73	65 ± 24.83	61.11 ± 21.18	0.556
Hospital stay, days	2 (2-2)	2 (2-2)	2 (2-2)	0.234
Other procedures than hysterectomy				
Bilateral salpingo-oophorectomy	38 (77.5%)	15 (68.1%)	23 (85%)	
Bilateral salpingectomy	11 (22.4%)	7 (31.8%)	4 (14.8%)	
Others	2 (4%)	1 (4.5%)	1 (3.7%)	
Intraoperative complications*	3 (6.1%)	3 (13.6%)	0	0.048
CTCAE 0-1	3 (6.1%)	3 (13.6%)	0	
CTCAE> 2	0	0	0	
Postoperative complications	3 (6.1%)	1 (4.5%)	2 (7.4%)	0.685
Clavien-Dindo grade 2	1 (2%)	0	1 (3.7%)	
Clavien-Dindo grade 3	2 (4%)	1 (4.5%)	1 (3.7%)	
Vaginal cuff closures exceeding 20 minutes	5/54 (9%)	4/26 (15.3%)	1/28 (3.5%)	0.134

## Discussion

Based on the findings of this study, there is no evidence that tridimensional HD vision during conventional laparoscopy significantly improves vaginal cuff closure time as compared to two- dimensional 4K HD vision systems. In 91% of cases, the GESEA 2 certified surgeons in training succeeded in completing the task. Additionally, there was no statistically significant difference in perioperative outcomes among the two groups in terms of EBL (p=0.556), HS (p=0.234), and postoperative complications (p=0.685). A significant occurrence of intraoperative complications in the 3D group (p=0.048) was shown. However, these were unrelated to the vision system or to the vaginal cuff suture: two were cases of vaginal wall lacerations during the transvaginal uterine retrieval related to the increased size of uterine fibroids, and one was a bladder superficial lesion when dissecting the vesicouterine plica made difficult by the presence of adhesions originating from a previous caesarean section. In one patient in the 4K group, a breakdown of the vaginal vault occurred in the first 30 days postoperatively following a traumatic event, which required surgical management. In 9% of cases, as the time taken for the vaginal vault closure exceeded 20 minutes, the task was completed by supervising surgeons so as not to have any harmful implications to patients due to the ongoing trial.

Currently, high-definition (HD) technology stands as the preferred choice for day-to-day laparoscopic surgeries. Alongside the conventional 2D HD, the ultra-high-definition (4K UHD) system, operating at a resolution of 3840 by 2160p, which is four times the pixels and double the resolution of HD, aims to leverage better image resolution and colour contrast for improved depth perception and outcomes. However, a notable drawback of two- dimensional laparoscopy lies in the absence of depth perception or stereoscopic vision. Although surgeons attempt to compensate for this limitation through indirect cues such as the relative size of objects, texture gradient, and motion parallax, these fall short of adequately replacing binocular depth vision (Pierre et al., 2009). This gap in depth perception prompted the introduction of tridimensional (3D) laparoscopic endovision systems. However, early iterations of 3D screens were associated with adverse effects such as visual strain, headaches, and nausea resulting in poor tolerance (Ukai et al., 2008). Limited and controversial data exist that directly compare conventional 2D to 3D laparoscopy in the context of gynaecological surgery. Studies in simulation settings suggest potential advantages with 3D vision as compared to 2D. A recent meta-analysis (Restaino et al., 2023) indicated that 3D imaging systems appear to enhance surgical performance for trainees, as compared to conventional 2D laparoscopy in studies involving a box trainer. The number of errors in the 2D laparoscopic group was significantly higher than in the 3D laparoscopic group for bimanual coordination (p < 0.00001) and suturing (p=0.007). However, in clinical studies, there was no significant difference in the time taken for total laparoscopic hysterectomy (p=0.44) and vaginal cuff closure (p=0.15) between the 2D and 3D groups (Restaino et al., 2021).

These controversies remain true even when specifically considering 4K systems (Harada et al., 2018; Kanaji et al., 2020). Indeed, available prospective trials on clinical settings have failed to demonstrate any differences between the 3D HD systems over the 4K HD ones in performing laparoscopic hysterectomies and cholecystectomies (Dunstan et al., 2020; Restaino et al., 2021) whereas Abdelrahman et al. (2018) on simulation tasks suggested advantages of the 4K HD system over 3D HD and 2D HD.

The diminished benefits observed in the clinical context of 3D may be attributed to a trade-off with the superior definition and high resolution of 4K. This enables enhanced anatomical discrimination, improved dissection, and overall surgical performance, all achieved without the drawbacks associated with 3D technology. Although a variety of new technologies are available, the best HD visual system is still to be identified and the benefits of 3D over 2D or 4K remain to be demonstrated. Despite the EAES recommendation to implement the adoption of 3D systems to save operating time (Arezzo et al., 2019), based on the findings of this study and in accordance with the published literature, it is not possible to statistically demonstrate this benefit in the gynaecological field. The strength of this randomised trial is its focus on surgeons in training and the originality of the subject matter. However, although a statistical sample calculation was performed before initiating the study, the enrolment of 54 patients in the absence of large differences and the variability introduced by the number of surgeons in training do not allow comprehensive conclusions to be drawn. Likewise, the results relating to the occurrence of intraoperative and postoperative complications must be read in the light of the sample size. Additionally, although the GESEA 2 certification does not demonstrate equal surgical competence or speed among trainees, it does demonstrate basic surgical skills as an additional guarantee to the surgical training completed by senior residents. It is critical to highlight that in 91% of cases, surgeons in training succeeded in completing the procedure in 20 minutes, demonstrating the ability to achieve complex laparoscopic tasks. Even if those data alone cannot demonstrate the efficacy of a training curriculum, they emphasise how practice and exercise on simulators for young surgeons in residency schools translate into good performance in the clinical practice.

The benefits of surgical certifications are evident and the need for structured education programmes which may combine theoretical sessions and dry lab with training in the operating rooms seem increasingly essential. Residency schools and leading surgical societies are working to establish mandatory training curricula for young surgeons. Certificates are endorsed by scientific societies to demonstrate the attainment of competence standards before surgeons may be considered autonomous in performing surgical procedures (Cardoso et al., 2023).

Suturing the vaginal vault stands out as one of the most complex tasks, primarily due to the required dexterity in intra-abdominal needle movements. Analogous to the historical enhancements in clinical outcomes witnessed during the shift from open to laparoscopic surgery—fuelled by the introduction of innovative vision systems and the gradual increase in proficiency for laparoscopic surgeons—the ongoing evolution of robotic platforms is anticipated to instigate a similar transformative effect (Lecointre et al., 2022).

Nearly all robotic-assisted surgery (RAS) systems now incorporate state-of-the-art 3D vision consoles, indicative of a trajectory favouring a substantial departure from 2D vision. Technological advances hold the promise of decreasing the surgeon’s side effects and augmenting spatial depth perception through increasingly superior image quality. Consequently, the learning curve for laparoscopic suturing, facilitated by the rotational range of motion of robotic instruments, is poised to shorten, potentially leading to a reduction in complications such as vaginal cuff dehiscence (Bradshaw and Advincula, 2009).

Additionally, training is on the point of a paradigm shift with the advent of robotics. It is due to the use of virtual systems in dedicated simulation consoles outside the operating room (OR) or in a dual-console set-up in the OR, empowering a collaborative approach where instruments can be controlled from each surgeon’s console by both experts and trainees (Simmonds et al., 2021).

In fact, currently robotic training pathways are not only offered by individual industries, but also endorsed by scientific societies alongside laparoscopic certifications (GESEA Robotics).

## Conclusion

There is no significant difference among 3D and 4K vision in operating time for vaginal cuff closure performed by trainee surgeons during conventional laparoscopy. The choice of surgical vision systems can be guided by a cost analysis and surgeon preferences. Structured training curricula and dry labs in university hospitals are extremely valuable for improving the skills of tomorrow’s surgeons.
